# Prion assemblies: structural heterogeneity, mechanisms of formation, and role in species barrier

**DOI:** 10.1007/s00441-022-03700-2

**Published:** 2022-11-18

**Authors:** Angélique Igel, Basile Fornara, Human Rezaei, Vincent Béringue

**Affiliations:** Université Paris-Saclay, INRAE, UVSQ, VIM, 78350 Jouy-en-Josas, France

**Keywords:** Prions, PrP, Transgenic mice, Quasispecies, Conformation, Autocatalytic

## Abstract

Prions are proteinaceous pathogens responsible for a wide range of neurodegenerative diseases in animal and human. Prions are formed from misfolded, ß-sheet rich, and aggregated conformers (PrP^Sc^) of the host-encoded prion protein (PrP^C^). Prion replication stems from the capacity of PrP^Sc^ to self-replicate by templating PrP^C^ conversion and polymerization. The question then arises about the molecular mechanisms of prion replication, host invasion, and capacity to contaminate other species. Studying these mechanisms has gained in recent years further complexity with evidence that PrP^Sc^ is a pleiomorphic protein. There is indeed compelling evidence for PrP^Sc^ structural heterogeneity at different scales: (i) within prion susceptible host populations with the existence of different strains with specific biological features due to different PrP^Sc^ conformers, (ii) within a single infected host with the co-propagation of different strains, and (iii) within a single strain with evidence for co-propagation of PrP^Sc^ assemblies differing in their secondary to quaternary structure. This review summarizes current knowledge of prion assembly heterogeneity, potential mechanisms of formation during the replication process, and importance when crossing the species barrier.

## Introduction

Prion diseases are a group of expanding fatal, infectious, neurodegenerative disorders affecting humans and wild-life or farmed animals. These diseases include Creutzfeldt-Jakob disease (CJD) in humans, scrapie in sheep and goats, bovine spongiform encephalopathy (BSE) in cattle, and chronic wasting disease (CWD) in a wide range of cervids (Collinge 2001; Prusiner 1998). Prion diseases have a worldwide distribution. In humans, they are considered as rare diseases; sporadic CJD has a worldwide incidence of 1.5 cases per year per million. In animals, the situation is more contrasted. Up to 12,000 cases of classical BSE (C-BSE) were reported each month in the UK at the peak of the “mad cow” epidemic in the early 1990s. In certain regions of North America, CWD is endemic, reaching a > 70% prevalence in captive herds (Moazami-Goudarzi et al. 2021). Classical scrapie, which is endemic in Europe, had prevalence estimates in sheep that varied from 0 (Portugal) to 245 ‰ (Cyprus) before implementation of control measures at the beginning of the twenty-first century (Fediaevsky et al. 2008). Prion diseases are constantly (re-)emerging. While circulating only in North America and for a limited period in Korea, CWD has suddenly emerged in Scandinavia in 2016 (Benestad et al. 2016) and is now threatening Europe (Hazards et al. 2019). A camelid prion disease has been discovered in Maghreb in 2018 (Babelhadj et al. 2018). In humans, the last identified prion disease is called variably protease-sensitive prionopathy (VPSPr). This very rare disease was discovered in 2008 (Notari et al. 2018).

Prions, the causative pathogens of prion diseases (Prusiner 1982), can propagate between different species. Prions have a zoonotic potential or are truly zoonotic agents, as exemplified by the emergence of variant CJD (vCJD) in humans due to the consumption of C-BSE contaminated food. There are currently great uncertainties about the exact number of vCJD asymptomatic individuals more than 20 years after emergence (Gill et al. 2020), because the molecular determinants of the species (or transmission) barrier that limits inter-species prion propagation remain mostly unknown.

Prions are formed from abnormally folded conformers (PrP^Sc^) of the cellular form of the prion protein (PrP^C^). In the mature form, PrP^C^ is a ~ 210-amino-acid-long, monomeric, glycosylphosphatidylinositol (GPI)-anchored membrane glycoprotein with high primary and tertiary structure identities across mammals. While PrP^C^ N-terminus is unstructured, PrP^C^ C-terminus is folded into a globular domain composed of three α-helices and a small two-stranded β-sheet (Eghiaian et al. 2004; Riek et al. 1996). PrP^C^ is ubiquitously expressed in the organism; the highest levels are found in the brain. PrP^C^ exerts a growing number of signaling functions in healthy individuals, from neuroprotection to stem cell biology (Halliez et al. 2015). PrP^C^ is also involved in other pathologies including cancer (Mouillet-Richard et al. 2021) and Alzheimer’s disease (Lauren et al. 2009). Abnormally folded PrP^Sc^ is enriched in β-sheet content and assembles into polydisperse amyloidogenic assemblies. Cryo-electron microscopy near-atomic-resolution structures of purified PrP^Sc^ are currently emerging; they suggest a reorganization of PrP^C^ in parallel in-register assembly within a fibrillar supra-organization (Kraus et al. 2021; Manka et al. 2022). PrP^Sc^ assemblies deposit mostly in the CNS. They also accumulate at variable levels, and in a strain-dependent manner, in many peripheral tissues, including notably the lymphoid tissue (spleen, lymph nodes, etc.).

As for conventional pathogens, PrP^Sc^ replicates and its biological activity or infectivity can be titrated, by bioassays in bioindicator animals or permissive cells or by cell-free amplification assays (Moudjou et al. 2020). PrP^Sc^ self-replicates by templating PrP^C^ conversion and polymerization. In sporadic cases, host PrP^C^ would, for unknown reason, misfold spontaneously into a replicative conformer initiating the self-replication process. In inherited cases, host PrP^C^ would misfold due to mutations in the PrP encoding gene (PRNP). In acquired cases, the initial PrP^Sc^ conformer would be acquired accidentally.

The quantitative aspects of prion accumulation (PrP^Sc^ levels or infectivity) during neuroinvasion have served to elaborate prion replication models. In essence, these models state that monomeric PrP^C^ is constantly recruited by PrP^Sc^ assemblies, allowing a cooperative production and accumulation of further PrP^Sc^ assemblies. The most popular models remain so far the autocatalytic conversion model by Griffith (1967) and the nucleated-polymerization model by Lansbury and Caughey (1995). While these two models describe qualitatively the cooperativity of PrP^Sc^ accumulation, they consider PrP^Sc^ assemblies as a static object. Moreover, they fail to describe the process of prion structural diversification and evolution. In this review, we show evidence that PrP^Sc^ assemblies are structurally heterogeneous. PrP^Sc^ heterogeneity will be considered at different scales, at the host population level with different circulating PrP^Sc^ conformations or strains but also within a single host and within a single strain. In the second part of the review, we will elaborate on the mechanisms of PrP^Sc^ diversification. In the third part of the review, we will discuss the importance of PrP^Sc^ heterogeneity in prion cross-species transmission.

## Structural diversity of PrP^Sc^ assemblies

PrP^Sc^ assemblies conformational heterogeneity must be considered at three different scales. At the population level, this diversity corresponds to the existence of different strains circulating in different hosts from the same species. Strains are conformational variants of PrP^Sc^ with specific biochemical and biological phenotypes. At the individual host level (i.e., field isolate), this corresponds to the coexistence of strains. One may be dominant and impose its phenotype but subdominant strains may co-propagate. At the strain level, this corresponds to different PrP^Sc^ subpopulations varying not only in their quaternary structures, but also at lower levels of structuration (tertiary and secondary), and impacting in turn their replicative and biochemical properties.

### Structural diversity of PrP^Sc^ assemblies at the host population level

In prion-susceptible host population, multiple prion strains are recognized due to structurally distinct PrP^Sc^ assemblies. Biochemically, these PrP^Sc^ species can differ in their posttranslational modifications (e.g., their relative ratio of glycoforms), their relative resistance to protease digestion or relative stability towards chaotropic treatment (e.g., urea or guanidine hydrochloride) or heat treatment (for review, see Beringue et al. 2008b). This points to profound differences at different structural levels. Phenotypically, in both infected hosts and in laboratory animals (notably transgenic mice expressing the mammalian PrP^C^ of interest), prion strains exhibit specific and synchronous incubation times, stereotyped clinical signs, and neuropathology, including the deposition of PrP^Sc^ and of vacuolar lesions in specified regions of the CNS, and specific tropism for peripheral tissues including the lymphoid tissue (Beringue et al. 2008b).

**Fig. 1:**
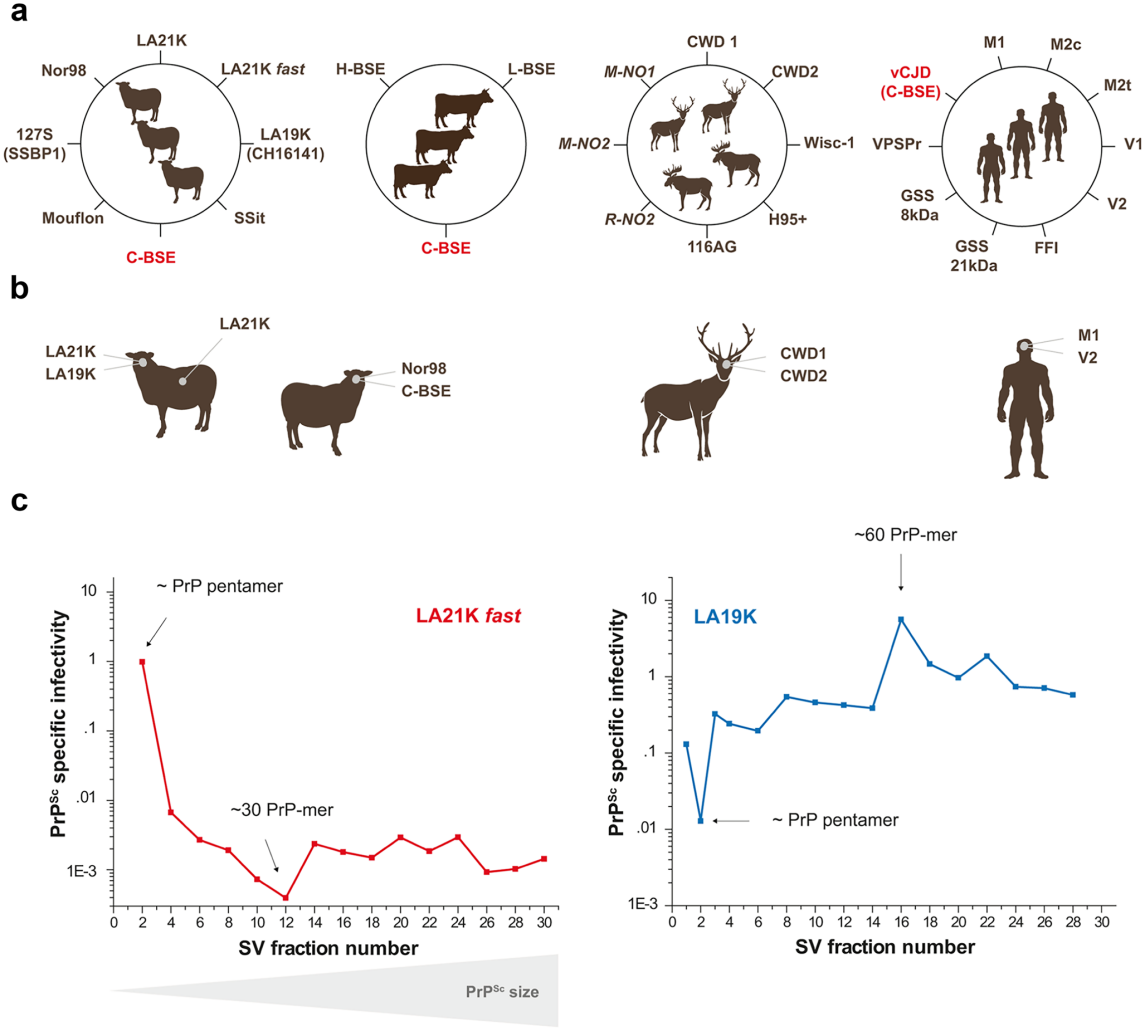
The many scales of PrP^Sc^ assemblies structural diversity. **a** Inventory of the major prion strains identified so far in permissive host species such as sheep, cattle, cervids, and humans. Prion strains are different PrP^Sc^ conformations with specific biological and biochemical attributes in infected host. In cervids, the strains in italic are specifically found in Scandinavia. In humans, the major CJD strains are grouped according to Bishop et al. categorization (Bishop et al. 2010) depending on PrP codon 129 genotype (M or V) and PrP^Sc^ electrophoretic signature (types 1 or 2). M1 strain regroups MM1 and MV1 cases, V2 strain regroups VV2 and MV2 cases, M2c and M2t strains correspond to MM2 cortical and thalamic forms respectively, and V1 strain corresponds to VV1 cases. Note that VV2 and MV2 may correspond to different strains (Jaumain et al. 2016). FFI, fatal familial insomnia; GSS, Gerstmann-Sträussler-Scheinker; VPSPr, variable protease-sensitive prionopathy. **b** At the single host level, different prion strains can co-propagate in the SNC or can co-propagate in distinct tissues, due to differential tropism for the lymphoid tissue. **c** Within a single strain, PrP^Sc^ heteroassemblies can be identified by sedimentation velocity (SV)–based size fractionation of PrP^Sc^ assemblies coupled with measure of specific infectivity. In this example, PrP^Sc^ assemblies originate from brains of terminally-sick ovine PrP transgenic mice infected with two biologically cloned scrapie strains replicating at different pace, LA21K *fast* and LA19K. The estimated size of the fractionated PrP.^Sc^ assemblies is indicated (data from Tixador et al. 2010)

Based on biochemical and neuropathological analyses in affected individuals and/or on strain typing studies in laboratory animals, at least eight different strains have been isolated from scrapie-infected sheep and goats (including atypical/Nor98 strain), three strains from bovines with BSE (the classical one responsible for the “mad cow” crisis (C-BSE) and two atypical, putatively sporadic strains), five strains from North American cervids infected with CWD, three strains from Scandinavian CWD, and ten strains from humans with prion diseases (Fig. 1a). This points to the fact that a given PrP^C^ primary structure—from a given host—can stably adopt different strain structural determinants (SSD) in the PrP^Sc^ state. Where the SSD are located in PrP^Sc^ assemblies and how the SSD specify different biological phenotypes remain poorly understood.

### Structural diversity of PrP^Sc^ assemblies at the host level

Within the same infected host, different strains (usually two) can co-propagate, at different rate. Many examples can be found in animal and human prion diseases. Transmission of classical scrapie field isolates from sheep and goats to transgenic mice expressing ovine PrP^C^ showed that a large proportion of the circulating isolates in Europe were composed of two co-propagating strains, which were termed LA19K and LA21K (Fig. 1b (Le Dur et al. 2017)). LA21K was dominant in most scrapie isolates imposing its biochemical phenotype (unglycosylated protease-resistant PrP^Sc^ fragment migrating at ~ 21 kDa by SDS-PAGE electrophoresis). To provide insights on the molecular determinants of strain predominance, sheep scrapie isolates containing variable proportions of LA21K and LA19K prions were transmitted experimentally to transgenic mice expressing ovine PrP^C^ at different levels in the brain. Remarkably, LA21K replicated dominantly in transgenic mice expressing near-physiological PrP^C^ levels, whereas LA19K phenotypically outcompeted LA21K in transgenic mice expressing high PrP^C^ levels (Le Dur et al. 2017). These experiments demonstrated that PrP^C^ levels can drive prion strain phenotypic dominance. They also suggested that local variations in native PrP^C^ levels—as observed in response to prion replication (Mays et al. 2014)—may favor the phenotypic selection of a given strain.

In the same infected host, co-propagating strains could preferentially replicate in distinct tissues. In the aforementioned example, LA19K replication was mostly limited to the CNS while LA21K colonized efficiently both the CNS and the lymphoid tissue (Fig. 1b (Beringue et al. 2020)). Such differential tropism may also be linked to different PrP^C^ levels, the spleen expressing ~ 20-fold reduced levels of the normal protein compared to the brain (Beringue et al. 2012).

In another example of co-propagating strains within a single host, the second co-propagating strain was barely detectable. Sheep infected with atypical scrapie (Nor98 strain (Benestad et al. 2003)) were found to co-propagate as minor strain C-BSE (Fig. 1b). C-BSE was identified following experimental transmission of atypical scrapie cases to transgenic mice expressing bovine PrP (Huor et al. 2019) or to pigs (Marin et al. 2021) and by in vitro prion amplification using a C-BSE optimized substrate. Remarkably, C-BSE prions were still detected in Nor98 prions that were biologically cloned by limiting dilution in ovine PrP transgenic mice, suggesting that Nor98 replication intrinsically co-generated C-BSE (Huor et al. 2019).

Transmission of North American CWD prions from diverse cervid sub-species to transgenic mice expressing deer PrP allowed isolation of two strain types, named CWD1 and CWD2. Both strains were found to co-propagate in deer (Fig. 1b (Angers et al. 2010)). Transmission of CWD from infected white-tailed deer to diverse bioindicator mice also lend support to the coexistence of CWD substrains; single polymorphisms at specific codons in the white-tailed deer PrP sequence could impact their relative fitness (Velasquez et al. 2020; Hannaoui et al. 2021).

In human, the co-detection in the brain of two PrP^Sc^ signatures termed type 1 and type 2 by immunoblot has been observed in many patients with sporadic CJD (Parchi et al. 1999; Polymenidou et al. 2005; Uro-Coste et al. 2008). Whether this co-occurrence is associated with the co-propagation of two strains or reflects the co-existence of PrP^Sc^ molecular subtypes with distinctive physicochemical properties is a debated issue. Recently, transmission of sporadic CJD cases to human PrP transgenic mice showed that each PrP^Sc^ signature was associated with a specific strain. In 80% of the investigated cases, the two strain types (M1 and V2) co-propagated in variable amounts (Fig. 1b (Cassard et al. 2020)).

To conclude, strain co-propagation in single infected host may be the rule rather than the exception. As co-propagation identification mostly relies on experimental transmission with more astringent replicative conditions for one of the strains, it cannot be excluded that strain co-propagation results from a unique progenitor strain that spontaneously evolves in these new conditions (see below) or because of intrinsic instability (Bruce and Dickinson 1987; Le Dur et al. 2017). Yet, in the aforementioned examples, there were stable manifestations of “pure” strains on serial transmission to the ad hoc bioindicator mice.

Co-propagation of strains in a single host raises the question of their genesis. Co-propagation is observed in acquired prion diseases, suggesting that multiple infection events could occur. It is also observed in sporadic cases, suggesting that multiple initiator events could occur or that the replication process allows generation of PrP^Sc^ assemblies with distinct SSD.

### Structural diversity of PrP^Sc^ assemblies at the strain level

#### Evidence for variably protease-resistant PrP^Sc^ assemblies

One of the most straightforward demonstrations of within-strain PrP^Sc^ heterogeneity is the existence of PrP^Sc^ conformers with markedly distinct susceptibility to proteinase K (PK) digestion. By developing a conformation-dependent immunoassay, which detects specifically PrP^Sc^ immunoreactivity versus PrP^C^ following chaotropic agent resolved denaturation, Safar and colleagues revealed the existence of protease-sensitive PrP^Sc^ alongside the canonical PK-resistant PrP^Sc^ conformer (termed PrP^res^). Protease-sensitive PrP^Sc^ was detected in eight different prion strains, including biologically cloned strains (Safar et al. 1998). Co-propagation of PK-sensitive and PK-resistant PrP^Sc^ subpopulations within a given strain was also shown with technics substituting PK by other proteases such as thermolysin (Cronier et al. 2008; Owen et al. 2007) or pronase (D'Castro et al. 2010). The exact contribution of PK-sensitive PrP^Sc^ conformers to prion physiopathology remains uncertain even if these species could represent up to 80% of total PrP^Sc^ content, specially at the early stage of prion replication. Gradual acquisition of PK-resistance by PrP^Sc^, as identified in kinetics studies, is suggestive of a dynamic involvement of PK-sensitive PrP^Sc^ in the replication process (Eskandari-Sedighi et al. 2021). At the terminal stage of the disease, depending on the experimental context, PK-sensitive PrP^Sc^ was found to be lowly infectious (Cronier et al. 2008) or highly infectious (Berardi et al. 2006) or be able to convert PrP in vitro (Pastrana et al. 2006). These seemingly contradictory observations highlight the fact that the replication process is intrinsically associated to structural diversification.

#### PrP^C^ post-translational modifications could affect PrP^Sc^ structuration PrP^Sc^ assemblies

Structural heterogeneity within a given strain may result from PrP backbone modification by prosthetic groups such as the GPI anchor or glycans. Single amino acid modification could conduce to strain mutation; therefore, it would not be surprising that variations in the number and nature of the prosthetic groups affect the conversion pathway and conduce to structural heterogeneity. For example, propagation of biologically cloned mouse prions in transgenic mice devoid of PrP GPI anchor affected their strain properties (Aguilar-Calvo et al. 2017; Mahal et al. 2012) or broadened their host spectrum (Race et al. 2015), suggesting profound structural differences between GPI-free and GPI-anchored PrP^Sc^ conformers. Remarkably, a minor proportion (~ 15%) of PrP^Sc^ is anchorless in wild-type animals (Stahl et al. 1990). Such presence may contribute to PrP^Sc^ conformational landscape during the replication process.

Glycans diversity may also contribute to further broadening of PrP^Sc^ structural landscape. PrP^C^ has two asparagine side chains linked to large oligosaccharides with multiple, diverse structures (Rudd et al. 2002). PrP^Sc^, as PrP^C^, is variably glycosylated at these two sites, which are located at amino acid positions 181 and 197 in the human PrP sequence (Endo et al. 1989). The stoichiometric ratio of PrP^Sc^ glycoforms is strain-specific and faithfully maintained during prion passaging in the same host species (Collinge et al. 1996; Somerville and Ritchie 1990), which means that a given strain has a specific preference for certain PrP^C^ glycotypes. Yet, PrP^Sc^ from different strains does not appear to differ in glycan composition (Nakic et al. 2021), suggesting that prion SSD are not encoded in glycans. However, PrP^Sc^ occupancy by glycans, given their extended size, variable proportion, and composition at each site (Nakic et al. 2021; Rudd et al. 2002), is likely to affect the stability, clearance and the dynamic of the forming assemblies by steric hindrance. Accordingly, transgenic modeling suggests that PrP^C^ glycosylation status can influence the efficacy of intra- and inter-species transmission of prions (DeArmond et al. 1997; Tuzi et al. 2008; Wiseman et al. 2015) and prion strain properties (Cancellotti et al. 2013). However, in cell-free prion amplification assays using unglycosylated PrP^C^ substrate, it was found that glycans were dispensable in specifying prion strain properties (Moudjou et al. 2016; Piro et al. 2009). These opposite results may be due to the different strains studied or to the point mutations inserted to prevent asparagine-linked glycosylation, because trafficking of the PrP mutant can be altered (Salamat et al. 2011) or because PrP post-translational state markedly influences the fate of the aggregates in the brain tissue (Sevillano et al. 2020).

#### Quaternary structure diversity of PrP^Sc^ assemblies, and beyond

Low-resolution structural studies such as sedimentation velocity (SV), size exclusion chromatography, and asymmetric fast-flow-field fractionation have been extensively used to explore the quaternary structure of PrP^Sc^ assemblies. In the brain of terminally sick animals solubilized in specific conditions, the size distribution pattern of PrP^Sc^ assemblies revealed the existence of subpopulations with distinct quaternary structure (Eskandari-Sedighi et al. 2021; Foliaki et al. 2019; Laferriere et al. 2013; Riesner et al. 1996; Silveira et al. 2005; Tixador et al. 2010). The analysis of different prion strains indicated that PrP^Sc^ quaternary structure pattern was strain-specific (Tixador et al. 2010).

When the specific infectivity or seeding activity values (i.e., the amount of infectivity or seeding activity per number of PrP) of size-fractionated assemblies were compared, the variations in the prion titer of the assemblies were decorrelated from size variations (extensively reviewed in Igel-Egalon et al. (2019a)). For example, in rapidly pathogenic scrapie prion strains, PrP^Sc^ assemblies with a size equal or below a PrP pentamer exhibited the highest specific infectivity values. These values were 1000 to 10,000-fold higher than those from the bulk of PrP^Sc^ assemblies with an estimated size of ~ 30-mers (Fig. 1c). Decorrelation was even more patent when other scrapie strains propagating at slower pace, such as LA19K or Nor98, were considered. Assemblies with the highest specific infectivity values were equivalent to ~ 30- to 60-mers of PrP. They were 1000-fold more infectious than PrP pentamers, which were the richest in terms of objects (Fig. 1c; Igel-Egalon et al. 2019a; Laferriere et al. 2013; Tixador et al. 2010)). Collectively, these data lend strong support to the view that the structural differences between size-fractionated PrP^Sc^ assemblies were not only due to quaternary structure variation but also involved variation in lower order structuration, at the tertiary and secondary level. These observations bring compelling evidence that a single prion strain is formed from a spectrum of structurally heterogeneous PrP^Sc^ subpopulations.

To conclude this section, there is compelling evidence that prions are formed from a spectrum of heterogeneous PrP^Sc^ assemblies. This pleiomorphism can be seen at different scales, in infected host populations, in infected hosts and within a single strain. Whether the intra-strain diversity is in fine similar to the inter-strain diversity remains to be seen. In the future, phenotyping tools with more discriminative power than bioassays may help resolving this question and identifying in PrP^Sc^ assemblies the domains responsible for intra- and inter-strain variability.

## Mechanisms of PrP^Sc^ structural diversification

### Theoretical considerations on the mechanisms of prion replication and structural diversification

**Fig. 2:**
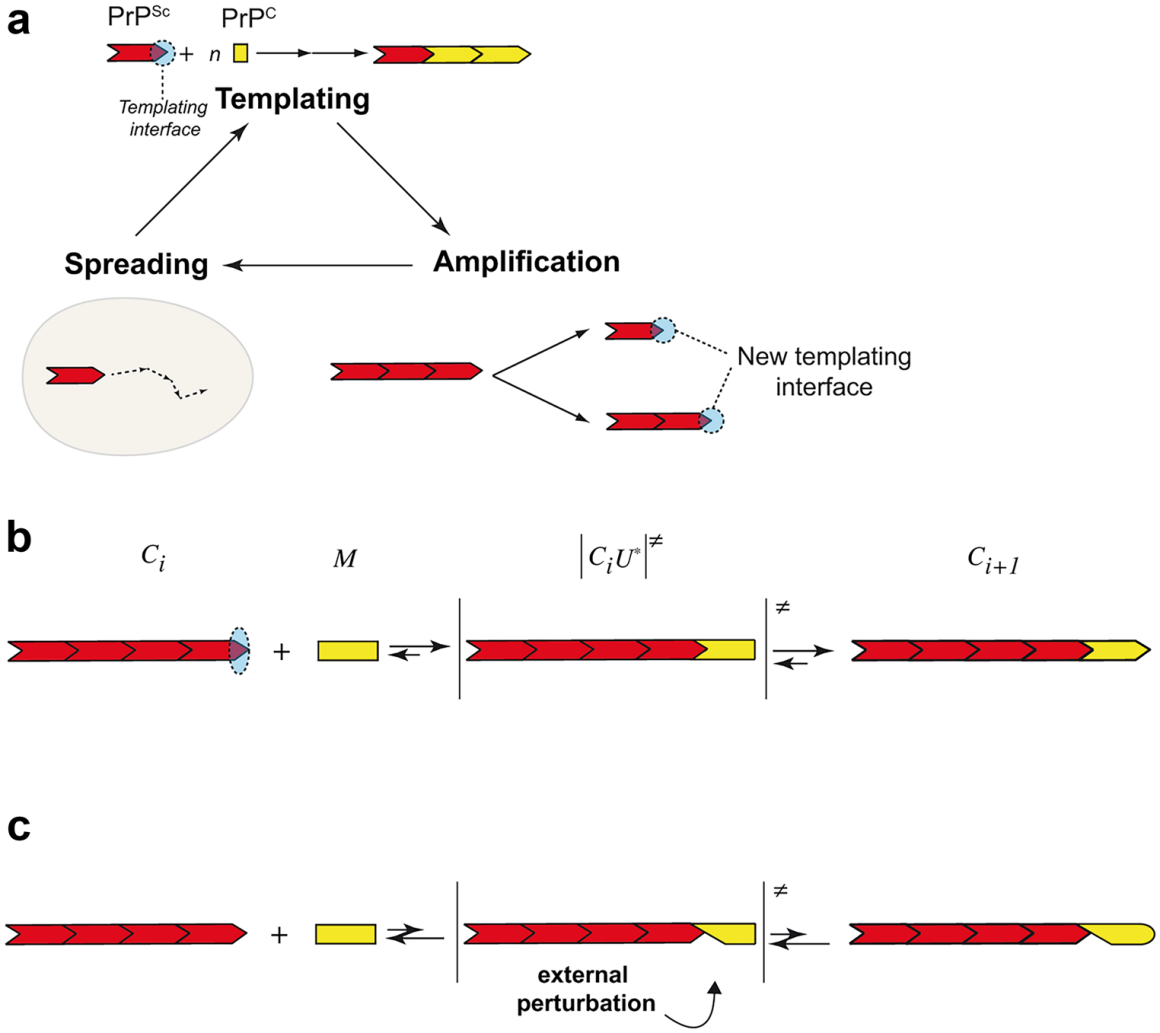
Prion replication mechanism. Schemes summarizing the current view of **a** the prion replication process and **b** the reaction mechanism of the end-elongation templating and induced fit adjustment of PrP^C^ on PrP^Sc^ assemblies. The prion replication process is a three-step process with templating, amplification, and spreading of the templating interface. The templating reaction mechanism can be divided in three elementary steps, each one being governed by an equilibrium between backward and forward steps. **c** An external perturbation can induce a misfit between PrP^Sc^ and PrP^C^, destabilize the complex, and displace the equilibrium toward the dissociation

The existence, in a single host, of different prion strains and, in a single strain, of PrP^Sc^ heteroassemblies, raises the question of such structural diversification. As a precise molecular mechanism of prion replication is lacking, only hypotheses can be formulated. The prion replication dogma, which remains mostly theoretical, can be split into three steps (Fig. 2a). The first step is the templating, during which PrP^Sc^ assemblies induce host PrP^C^ conformational change through the templating interface. The asymmetric evolution of the templating process (PrP^Sc^ is converting PrP^C^ and not the opposite) is only due to the higher stability of PrP^Sc^ assemblies compared to the stability of the PrP^C^ fold. The amyloid end-elongation by monomer addition remains until now the most widely accepted mechanism of templating (Collins et al. 2004). In this process, amyloid fibril ends would serve as templating interface for monomeric PrP^C^ and induce its structural rearrangement by a mechanism resembling the induced fit adjustment or conformational selection (Csermely et al. 2010; Koshland 1963). In the end-elongation hypothesis, the number of templating interface remains constant. Thus, an amplification step is required to accommodate the exponential aspect of prion replication (Langevin et al. 2011; Nakaoke et al. 2000). The second step is the amplification of the templating interface, putatively by an assisted fragmentation (Shorter and Lindquist 2006). The third step, responsible for prion dissemination through the infected tissue is the spatial spreading of the templating center. One can easily conceive a stochastic formation of structurally distinct sets of assemblies from different states of prionogenic monomeric PrP^C^. It is harder to physico-chemically conceive how, during the templating step, structural diversification could take place without any external perturbation (thermal and environmental fluctuations of the PrP^C^ protein) or change in PrP^C^ backbone and/or post-translational modifications. Yet, such external perturbations have to accommodate the reaction mechanism at work in the end-elongation templating. The induced fit adjustment of PrP^C^ on PrP^Sc^ can be decomposed in three elementary steps, each one being governed by an equilibrium between backward and forward steps (Fig. 2b). In the first step, PrP^Sc^ assemblies will interact with PrP^C^ through single or multiple specific interaction interfaces. This interface could be strain-specific. The N-terminal, polybasic region of PrP^C^ (residues 23–31 (Turnbaugh et al. 2012)) and regions containing residues 89–112 and 136–158 (Moroncini et al. 2004; Solforosi et al. 2007) have been reported to be involved in the interaction between PrP^Sc^ and PrP^C^. The second step, which is concerted with the formation of the PrP^Sc^-PrP^C^ complex, consists in an (at least partial) unfolding of PrP^C^ into PrP^U*^. Indeed, among all amyloidogenic proteins, PrP^C^ stands apart amid its folded native state (Eghiaian et al. 2004; Riek et al. 1996). Therefore, the templating process inducing PrP^C^ structural transition to PrP^Sc^ should first disrupt at least some interactions existing in the native fold (i.e., partial unfolding) prior to the acquisition of any new fold. The importance of this step is experimentally well illustrated in the cell-free formation of synthetic prions where a partial unfolding of recombinant PrP is required to induce PrP fibrils formation (Makarava et al. 2011). It is not straightforward to figure out the impact of external perturbations on these elementary steps. Indeed, as any reaction process—be it irreversible or not—can be decomposed into multiple micro-equilibria, a misfit between PrP^Sc^:PrP^C^ will destabilize the complex and displace the equilibrium toward the dissociation (Fig. 2c).

To conclude, it is difficult to consider a physico-chemically relevant PrP^Sc^ diversification process in the frame of the current, theoretical prion polymerization mechanism.

### PrP^Sc^ assemblies diversification due to prion replication

**Fig. 3:**
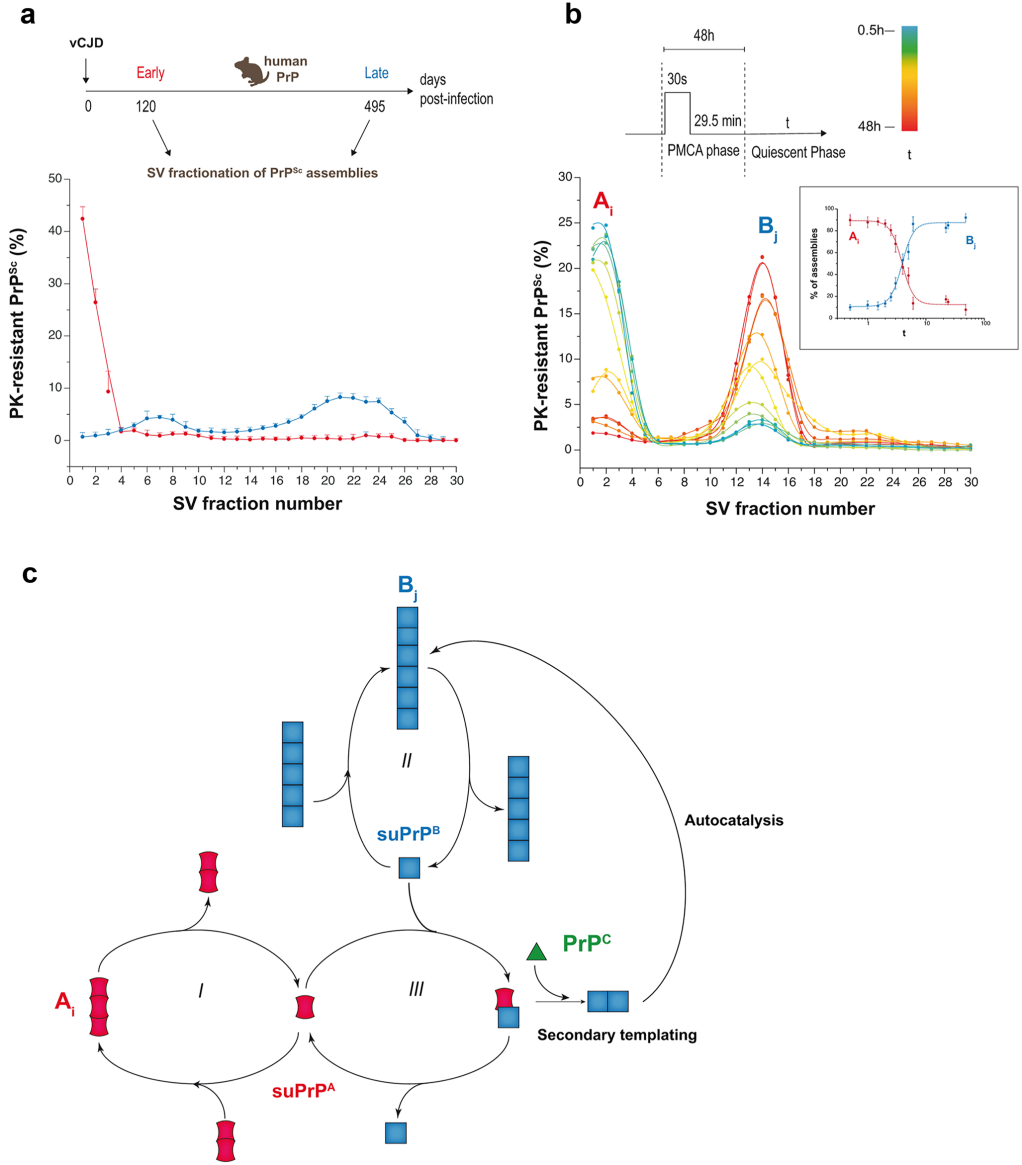
PrP^Sc^ diversification during prion replication process by PMCA. **a** SV-based size distribution of PrP^Sc^ assemblies at early and late stages of vCJD prion pathogenesis in the brain of human PrP mice. **b** SV-based PrP^Sc^ size distribution in PMCA products (127S scrapie strain) analyzed immediately at the end of the PMCA reaction or after further quiescent incubation at 37 °C for the indicated period of time (t). This allows identifying two populations of PrP^Sc^ assemblies termed *A*_i_ and *B*_j_ (with i < j). *A*_i_ is mostly generated during the active phase of the PMCA reaction. During the quiescent phase, *A*_i_ decreases over time in favor of *B*_j_ according to a bimodal process (without appearance of assemblies of intermediate size). The inset graph shows the variations of the percentage of *A*_i_ and *B*_j_ as a function of the quiescent phase (t). The sigmoidal form of the curves is indicative of an autocatalytic reaction process. **c** Kinetic scheme describing the transformation process of *A*_i_ into *B*_j_. *A*_i_ and *B*_j_ are in equilibrium with their respective suPrP (Igel-Egalon et al. 2017; steps I and II). The best model to account for the cooperative, PrP^C^-dependent transformation of A_i_ into B_j_ implicates the formation of a complex between suPrP^A^ and suPrP^B^ (step III). This complex is at the origin of the secondary templating pathway whereby the transformation of suPrP^A^ to suPrP^B^ is assisted by suPrP^B^, making the process autocatalytic (data from Igel-Egalon et al. 2019b)

A change of paradigm is necessary to accommodate PrP^Sc^ diversification with the prion replication mechanism. To achieve progress on this issue, we studied kinetically prion assemblies structural diversification during prion replication (Igel-Egalon et al. 2019b). The size distribution analysis (by SV) of PrP^Sc^ assemblies from three distinct cloned prion strains (human vCJD, mouse 139A, and scrapie 127S prions) at different time points of the disease showed that the early steps of prion replication generated small oligomeric PrP^Sc^ objects (Fig. 3a). The formation of larger-size PrP^Sc^ assemblies appeared to be a secondary step in the evolution of the disease and was concerted with the disappearance of the small oligomeric PrP^Sc^ objects. The imprecision and the variability of the in vivo experiments required the exploration of the early stage of prion replication through an in vitro bona fide prion replication system such as protein misfolding cyclic amplification (PMCA (Saborio et al. 2001)).

In the PMCA assay, minute amounts of PrP^Sc^ are mixed with a substrate containing PrP^C^. Usually, this substrate is a transgenic mouse brain homogenate or a cell lysate expressing the mammalian PrP^C^ of interest (Moudjou et al. 2016, 2014). The mixture is then submitted to alternative cycles of sonication/quiescent incubation at 37 °C for 1–2 days. Many rounds can be done by refreshing the PrP^C^ substrate. PMCA mimics the prion replication process as, most often, it generates high levels of prion infectivity and maintains prion strain biological properties (Castilla et al. 2008a, b; Moudjou et al. 2016, 2014). As mentioned above, SV coupled with measure of specific biological activity of the fractionated assemblies allows isolating different structural states of PrP^Sc^ in the brain of terminally-sick mice. Applying this strategy to PMCA generated prions showed that the prion replication process generated two subsets of structurally different PrP^Sc^ assemblies. Their process of formation was intricated and sequential, regardless of the strain considered (Igel-Egalon et al. 2019b). For the three different cloned prion strains studied in vivo (human vCJD, mouse 139A, scrapie 127S), the first replication step generated mostly small PrP^Sc^ oligomers (termed *A*_i_). *A*_i_ size was below a PrP pentamer. The second step, which required the presence of PrP^C^, transformed the *A*_i_ oligomers into larger ones (termed *B*_j_) (Fig. 3b). This was accompanied by further structural rearrangement at the level of the secondary/tertiary structure, as identified by (1) differences in *A*_i_ and *B*_j_ specific infectivity values, (2) the irreversibility of the transformation of *A*_i_ into *B*_j_ (i.e., *B*_j_ is not an *A*_i_ condensate), and (3) the structural differences in the elementary bricks (Igel-Egalon et al. 2017) constitutive of *A*_i_ and *B*_j_ assemblies. Kinetic studies and mathematical modeling showed that the transformation process of *A*_i_ into *B*_j_ assemblies through this secondary templating pathway was cooperative and under the control of PrP^C^ substrate consumption (Fig. 3c). Collectively, these data lend support to the view that the prion replication process is intrinsically a source of PrP^Sc^ assemblies diversification within a single strain. This occurs in a deterministic manner, thus contradicting common belief supporting stochastic events or environmental fluctuations at the origin thereof (Weissmann et al. 2011).

To conclude this section, there are at least two possible main ways for prion structural diversification, one due to the replication process itself and the other one due to PrP^C^ itself, under the influence of environmental fluctuations where replication occurs.

## PrP^Sc^ diversification and prion adaptation

### The species barrier phenomenon

**Fig. 4:**
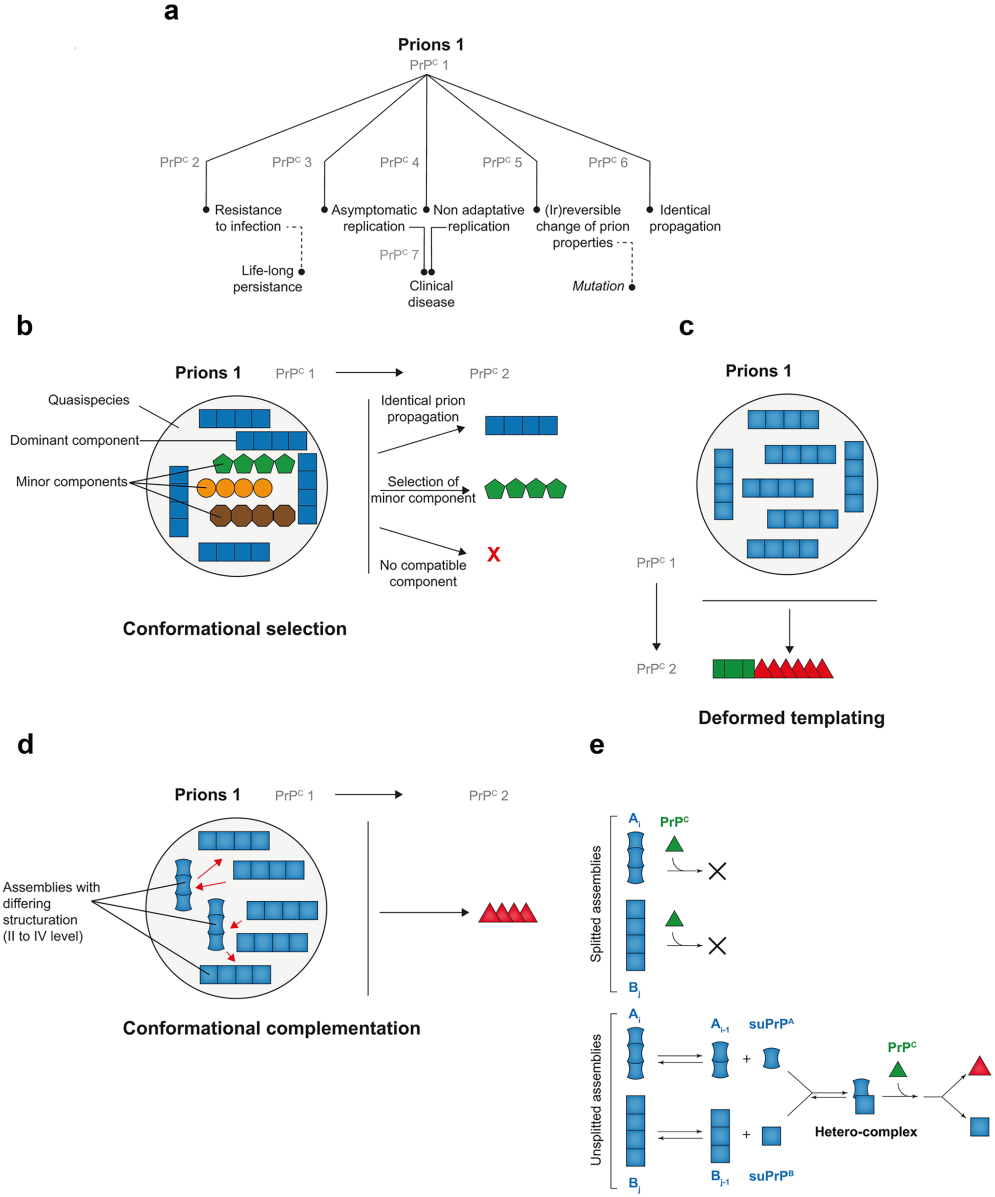
Molecular determinants of prion species barrier. **a** Main outcomes observed during experimental prion cross-species transmission (or transmission to heterologous PrP^C^ as done by transgenic modeling). To be pointed out, cross-species transmission of a different prion from species/PrP^C^ 1 may lead to drastically different outcomes. **b** Conformational selection model to explain prion cross-species transmission at the molecular level. In this model, prions would be composed of a cloud of subcomponents or substrains in varying proportions. The major component would be responsible for the strain phenotype in the parental host species. Other substrains would be co-propagated as minor components. On cross-species transmission, the optimized subcomponent, i.e., the component that lies within the portfolio of acceptable conformations in the new PrP^C^ species, would be preferentially selected. The issue of the transmission will thus mostly depend on the presence of a compatible component and relative concentration. **c** In the deformed templating model, prion primary passage to species expressing heterologous PrP^C^ would be inefficient, because of structural incompatibility between infecting PrP^Sc^ and PrP^C^. This would lead to generation of PrP^Sc^ with atypical conformation (green square) in a reduced number of asymptomatic animals. On subpassage, this conformation would slowly evolve toward an optimized conformation (red triangle), allowing adaptation. **d–e** In the conformational complementation model (**d**), prions would be composed of heterogeneous PrP^Sc^ assemblies with respect to secondary, tertiary, and quaternary structure. Interaction between these assemblies (red arrows) would allow crossing the species barrier. Mechanistically (**e**), the complex formed by the suPrPs from the different PrP^Sc^ assemblies (here *A*_i_ and *B*_j_) would provide an interaction interface with heterologous PrP^C^ that is absent in each assembly or each individual suPrP

Prions can transmit between different species. Yet, this capacity can be restricted by a so-called species or transmission barrier. Prion cross-species transmission issues are highly variable (Fig. 4a). Two extreme issues are well documented; transmission can occur with little or no species barrier (“faithful” or “identical” transmission) or be negative (e.g., Nonno et al. 2006)). In the last case however, prions may persist in the brain for the entire life of the inoculated host because of their pronounced resistance to clearance (Martin et al. 2021). Most often, prion cross-species transmission is difficult, necessitating serial passaging in the new host to adapt and reach 100% attack rate with stabilized disease tempo and consistent neuropathological or biochemical phenotypes. In certain cases, a new prion strain type may suddenly emerge (usually from the second passage onwards), as shown by a drastic reduction of the incubation time to disease and stabilized phenotype (e.g., Chapuis et al. 2016). Usually, this new strain type has lost the capacity to reinfect the parental host. This phenomenon is termed prion “mutation” by analogy with conventional pathogens. There are also reports of prions able to replicate on primary passage in animals expressing PrP^C^ from a foreign species but unable to adapt on further passage, a process referred to as nonadaptive prion amplification (Bian et al. 2017; Duque Velasquez et al. 2020). In short, when prions are confronted to a new host or a new PrP^C^ sequence, all outcomes are seemingly possible.

### Host determinants of the species barrier

Prion cross-species transmission outcome is critically dependent upon host–pathogen interactions. From the host side, the route of infection and the gene encoding PrP are two critical determinants.

During intraspecies transmission events, the efficacy of infection can vary by a million-fold depending on the route of inoculation (Haybaeck et al. 2011; Herzog et al. 2004; Kimberlin and Walker 1978, 1988; Lasmezas et al. 2005, 2001; Taylor et al. 1996). This route-dependent efficacy has been extrapolated to interspecies transmission events, but to our knowledge, no systematic assessment of the minimal infectious dose relative to the route of inoculation has been done.

Transgenic modeling by Prusiner’s group demonstrated that PrP primary structure homology between the host and the infecting prion was sufficient to abrogate prion species barrier. Hamster Sc237 prions which do not induce a clinical disease in wild-type mice propagated readily in transgenic mice expressing hamster PrP (Scott et al. 1989). This seminal experiment paved the way for transgenic mouse models that abrogate prion species barrier in laboratory animals. As another example, sporadic CJD prions, which do not replicate in wild-type mice, faithfully propagate in transgenic mice expressing human PrP (Asante et al. 2002; Beringue et al. 2008a; Collinge et al. 1995). It was later shown that PrP sequence homology is not a prerequisite as sporadic CJD prions replicate in bank vole (*Myodes glareolus*) with little or no species barrier (Nonno et al. 2006). Yet, bank vole and human PrPs share a 90% amino acid sequence identity. Bank voles or transgenic mice expressing bank vole PrP^C^ may be universal prion acceptors because of their capacity to propagate many strains from many different species (Watts et al. 2014), including prions reputedly difficult to transmit such as those responsible for VPSPr or Gerstmann-Sträussler-Scheinker syndrome (Nonno et al. 2019; Pirisinu et al. 2016).

The fact that expressing PrP with a sequence identical to that of the infecting prion most often if not always abrogates the species barrier in mice or in other, potentially less permissive species like rabbit (Sarradin et al. 2015) or drosophila (Thackray et al. 2018), lends support to the view that they are no non-PrP genes essential to prion cross-species transmission.

It must be noted that within the same species, PRNP polymorphisms can modulate the disease susceptibility to a degree of magnitude like that observed in interspecies transmission event. For example, sheep expressing the ARR allele at PRNP codons 136, 154, and 171 instead of the ancestral ARQ allele (where A, R, and Q stand for alanine, arginine, and glutamine, respectively) are highly resistant to classical scrapie prions (Elsen et al. 1999). In human, a naturally occurring variant at PRNP position 127 (valine instead of glycine) protects against prions responsible for CJD or kuru, an acquired form of CJD due to cannibalistic rituals (Asante et al. 2015). In human, the common polymorphism at PRNP codon 129, where either methionine or valine is present, provides a relative protection, notably against clinical forms of vCJD (Fernandez-Borges et al. 2017; Wadsworth et al. 2004). In cervids infected with CWD, PrP^C^ polymorphisms emerge as important driver of prion selection and evolution, particularly in heterozygous animals (Velasquez et al. 2020; Hannaoui et al. 2021).

### Pathogen determinants of the species barrier

From the pathogen side, the prion strain type and obviously the dose inoculated are two critical determinants, the dose being itself critically related to the route of infection.

As mentioned above, strains responsible for the different forms of sporadic CJD do not replicate in wild type mice, despite inoculation at high dose and intracerebral inoculation. Oppositely, vCJD prions can propagate in these animals, the force of the species barrier depending on the genetic background and the associated polymorphisms in the PrP-encoding gene (Bruce et al. 1994).

Strain-dependent susceptibility is similarly observed for the PRNP polymorphisms within the same species. Thus, sheep expressing the ARR allele become fully susceptible to the Nor98 strain responsible for atypical scrapie (Le Dur et al. 2005).

### The species barrier is tissue-specific

The capacity of invading prions to replicate extraneurally in the newly infected host is critical as foreign prions can establish easier in the spleen tissue than in the CNS (Beringue et al. 2012; Bian et al. 2021). Transgenic modeling showed that the spleen was 7–tenfold leakier than the brain to prions that adapted with difficulty to foreign PrP species. This included notably C-BSE prions in human PrP transgenic mice expressing methionine at codon 129. Such leakiness of the spleen versus the brain may explain why the number of clinical cases of vCJD is limited, while the number of asymptomatic individuals with PrP^vCJD^-positive lymphoid tissue is high (Collinge 2012; Gill et al. 2020).

### Molecular aspects of the species barrier and prion assemblies diversification

The aforementioned examples indicate that the force of prion species barrier depends on the possibility of interactions between tissue-specific PrP^C^ and the infecting prion strain type. At the molecular level, it is believed that constrained steric interactions between PrP^C^ and PrP^Sc^ are the limiting factor. How could this view accommodate PrP^Sc^ assemblies diversity? It may be anticipated that the larger the diversity of PrP^Sc^ assemblies structures, the greater the probability of interactions with foreign PrP^C^.

#### The conformational selection model

Before PrP^Sc^ heterogeneity was even unraveled at the strain level, but rather by analogy with the (viral) quasispecies concept, Collinge and Clarke (2007) adapted the conformation selection model (Csermely et al. 2010; Tsai et al. 1999) to explain prion cross-species transmission outcome at the molecular level (Fig. 4b). In essence, this model posits that (i) prions are not clonal but constitute an ensemble with dominant and subdominant PrP^Sc^ components or substrains and (ii) host PrP^C^ can accommodate a certain portfolio of PrP^Sc^ conformations in the pathological state. On cross-species transmission, if one (major or minor) subcomponent lies within the portfolio of conformations host PrP^C^ can accommodate in the PrP^Sc^ state, there will be preferential selection of this compatible conformation and crossing of the species barrier. It is thus expected that the time to disease onset and the attack rate in the new host will mostly reflect the initial concentration of this optimized subcomponent. Above a certain concentration threshold, the attack rate should be relatively high.

Regarding PrP^Sc^ assemblies molecular diversity, this model would readily accommodate prion strains co-propagation in a single host, one being preferentially selected on cross-species transmission amid a compatible conformation. It might potentially accommodate PrP^Sc^ assemblies heterogeneity at the intra-strain level, as long as one consider that these assemblies can become bona fide strains in the new host. The conformational selection model does not address the molecular mechanism for mutant emergence as such mutant cannot pre-exist in the quasispecies of molecular substrains per se (otherwise, it would be readily selected). The model states in essence that mutation can occur because of the intrinsic instability of certain strains (Collinge 2016).

#### The Deformed templating model

The deformed templating model is the second model to explain prion species barrier at the molecular level (Fig. 4c). It stems from the difficulty of certain minimalistic preparations of recombinant PrP fibrils to transmit disease to bioindicator animals (Makarava and Baskakov 2013; Makarava et al. 2011, 2016). In these experiments, following recombinant PrP fibrils inoculation, “atypical” forms of PrP^Sc^ accumulated in few asymptomatic animals on primary passage. On further passage, virulence gradually increased and “classical” PrP^Sc^ species emerged. The deformed templating hypothesis (Makarava et al. 2011) that stems from these results is based on an end-elongation replication process. It considers recombinant PrP assemblies as highly homogenous. It also considers that because bacterial PrP does not express glycans and the GPI anchor, it creates a structural barrier on contact with mammalian PrP^C^ which expresses these post-translational modifications. With these hypotheses, the first step of the cross-species transmission will consist to create a certain degree of heterogeneity on primary passage. After this heterogenization step, the authors made the hypothesis that with several cycles of templating, the templating interface will progressively shift to a more efficient templating interface. This two-step process tentatively explains how a strong species barrier could be crossed and may explain the emergence of prion mutant. Yes, it suffers several limitations. First, other preparations of recombinant PrP fibrils are directly highly infectious, without occurrence of PrP^Sc^ structural shift (Choi et al. 2016; Legname et al. 2004). Second, it does not consider PrP^Sc^ assemblies diversity. Third, the slow, progressive evolution of the templating interface over passaging should be put in perspective with the number of PrP^Sc^ templating events at each passage (approximately 10^14^ replicating events for hamster 263 K (Igel-Egalon et al. 2017)).

#### The conformational complementation model

**Fig. 5:**
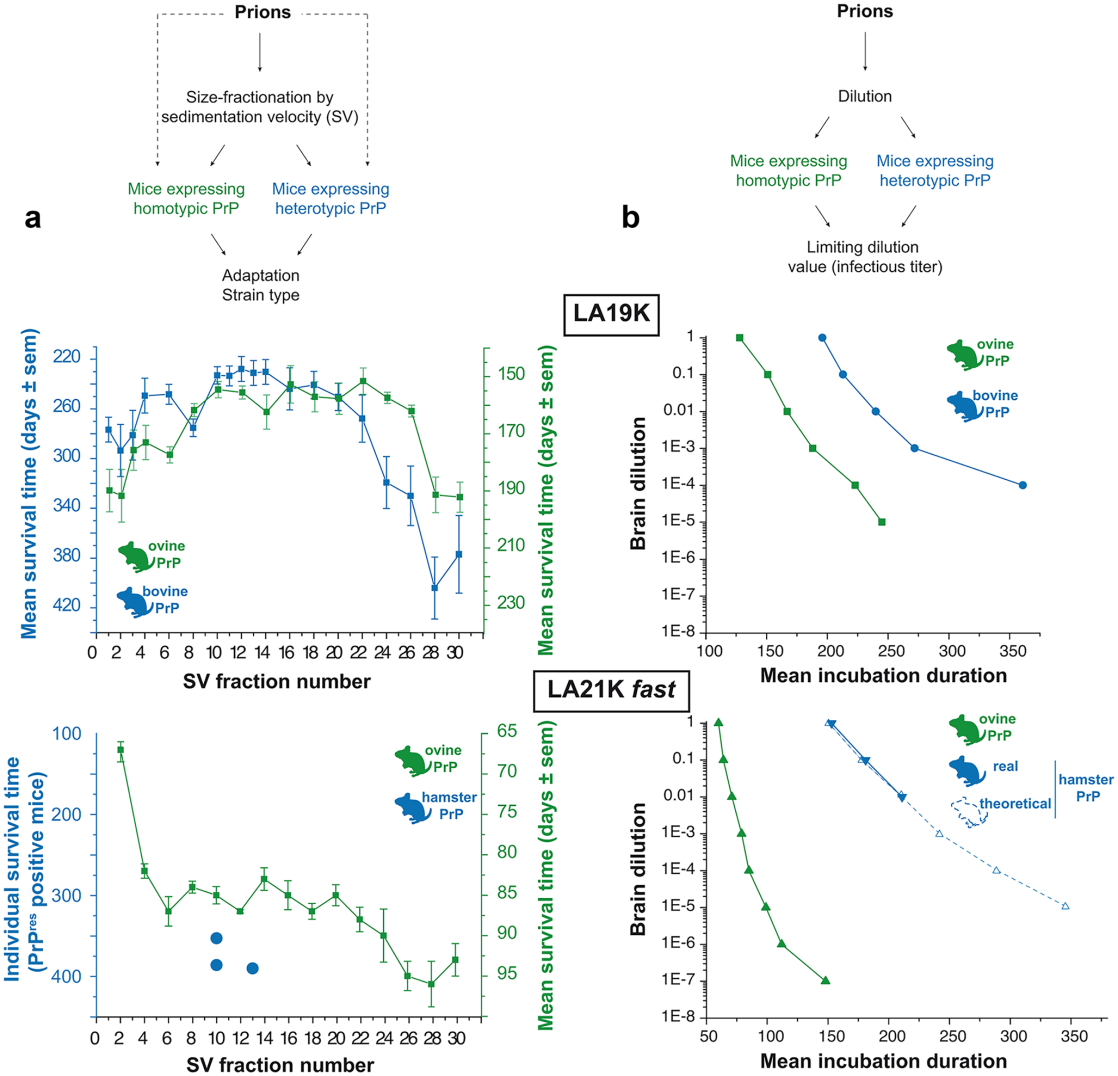
Effects of fractionating PrP^Sc^ assemblies by sedimentation velocity or dilution on prion capacity to cross the species barrier. The cloned scrapie prion strain LA19K propagates without any apparent species barrier from ovine PrP transgenic mice to bovine PrP transgenic mice. The cloned scrapie prion strain LA21K *fast* can adapt to hamster PrP transgenic mice; achieving a stable biological phenotype in these mice necessitates several passages, indicative of a substantial species barrier. **a** Effect of SV fractionation on the capacity of LA19K and LA21K *fast* prions to propagate in heterologous PrP^C^ mice. This was not detrimental to LA19K prions with respect to the disease incidence and size distribution of infectivity (inversely correlated to the mean incubation time shown here) as compared to the original host PrP^C^. On the opposite, LA21K *fast* priogenesis was almost completely abrogated with only 3 mice found asymptomatic out of the 300 analyzed (Igel-Egalon et al. 2020). **b** Effect of dilution on the capacity of LA19K and LA21K *fast* prions to propagate in heterologous PrP^C^ mice. The limiting dilution values achieved with LA19K prions were similar in the homologous and heterologous PrP^C^ contexts (top panel, compare green and blue lanes). Note that for LA19K in bovine PrP mice, the 10^−5^ dilution was not tested. At the 10^−4^ dilution, two-thirds of the mice were affected, as for the 10^−5^ dilution in ovine PrP mice. On the opposite, the limiting dilution values achieved with LA21K *fast* were reduced by 10,000-fold in the heterotypic PrP^C^ context (bottom panel, compare blue and green plain lanes). In theory (blue dotted lane), the limiting dilution value in the heterotypic context should have been 1000-fold higher for LA21K *fast*, as calculated from the attack rate and disease tempo obtained at low dilution and extrapolation from other bioassays (Igel-Egalon et al. 2020). There was thus a strong impact of the dilution on LA21K *fast* capacity to cross the ovine to hamster transmission barrier

The conformational complementation model is the only model that considers the structural diversity of infecting PrP^Sc^ assemblies. In addition, it implicates the existence of synergetic interactions between these differing subpopulations (or a component thereof) (Fig. 4d). This model stems from experiments where the importance of PrP^Sc^ assemblies structural diversity in cross-species transmission events was specifically addressed (Igel-Egalon et al. 2020). PrP^Sc^ assemblies were separated from each other either by SV-based size-fractionation or by serial dilution before transmission to transgenic mice expressing a foreign PrP^C^ sequence. In the absence of a transmission barrier, separating or diluting PrP^Sc^ assemblies was without influence on the disease tempo and prion strain properties (Fig. 5a, b, top graph). In the presence of a species barrier, fractionating PrP^Sc^ assemblies by SV overtly delayed and even abrogated priogenesis (Fig. 5a, bottom graph), despite sufficient infectivity load of the isolated assemblies to adapt per se. Dilution had also a severe impact, the efficacy of infection being 10,000-fold decreased compared to the homotypic PrP context or 1000-fold decreased compared to the expected value in the heterotypic PrP context (Fig. 5b, bottom graph).

It could be argued that these experiments are congruent with the conformational selection model, the loss of subcomponents by fractionation or dilution resulting in the loss of optimized conformations in the heterotypic PrP context, and thus in delaying/abrogating priogenesis. A first counterargument is that the isolated PrP^Sc^ assemblies that finally adapted on serial passage in the new PrP transgenic host did not differ in terms of strain properties from unfractionated prions, meaning that if an optimized conformation pre-existed, it was not lost during fractionation. A second counterargument necessitates to detail one of the experimental paradigms used: The rapidly pathogenic LA21K *fast* scrapie prion strain (Fig. 1c) was fractionated before transmission to transgenic mice expressing hamster PrP. The pentameric oligomers with the highest specific infectivity values (Figs. 1c and 5a) in the homotypic context had sufficient infectivity levels in the heterotypic context to adapt. They did not elicit a clinical or subclinical disease. The larger assemblies with the lowest specific infectivity values in the homotypic PrP context elicited a subclinical disease in a very low proportion of mice. It could thus be said that these assemblies had the optimized conformation for the hamster PrP sequence. Yet, because there are by far the most populous conformation in the unfractionated prion strains (Tixador et al. 2010), they should have elicited the disease at higher attack rate because of their presence in substantial amounts in the SV fractions.

As a simple selection of optimized PrP^Sc^ conformations is unlikely to explain these observations, we thus posited that somehow the assemblies should complement each other to cross the species barrier, i.e., to interact with foreign PrP^C^. How is complementation mechanistically possible? In its simplest acceptation, it implicates interactions between heteroassemblies to create a new structural information, absent in each single assembly, that allows interaction with heterologous PrP^C^ (Fig. 4e). As mentioned above, structurally distinct PrP^Sc^ assemblies are generated by the prion replication process, i.e., *A*_i_ and *B*_j_ with their specific subunits suPrP^A^ and suPrP^B^. Mechanistically, we found that the secondary templating pathway that generates, in a PrP^C^-dependent manner, B from A, involves the formation of a suPrP^A^/suPrP^B^ heterocomplex (Fig. 3c (Igel-Egalon et al. 2019b)). This is possible because *A*_i_ and *B*_j_ are constitutively in dynamic equilibrium with their own suPrP (Igel-Egalon et al. 2017, 2019a). The suPrP^A^/suPrP^B^ heterocomplex may interact with heterologous PrP^C^ because it may have a templating interface that is not present in suPrP^A^ and suPrP^B^ due to the structural constraints imposed by the interactions. On interaction with heterologous PrP^C^, such a new templating interface would lead to the formation of a de novo suPrP^B*^ with an optimized templating interface for further conversion. The force of the species barrier would thus depend on the stability of the heterocomplex, the possibility of a new interface with heterologous PrP^C^ and on PrP^C^ concentration for the cooperativity of the reaction. This complementation model would best accommodate the within-strain PrP^Sc^ assembly diversity and the underlying dynamic mechanism of genesis.

To conclude this section, PrP^Sc^ assemblies diversity at the level of the host or the strain must be taken into account when addressing at the molecular level the issue of prion evolution during cross-species transmission. The conformation selection models and the complementation models consider PrP^Sc^ diversity with respect to prion substructure or substrains. The second model would go a step further by taking account the dynamics of the assemblies.

## Conclusions

This review tentatively unravels the many shades of prion assemblies diversification and the need to bypass the “one assembly fits all” approach to understand prion replication and adaptation at the molecular level. PrP^Sc^ assemblies diversity, dynamic of formation, and exchange provide new mechanistic insights into prion replication and adaptation. This provides prions with an evolutionary advantage due to selection of best replicator or mutational events in different environments to finally ensure persistence and diffusion within the host or at the population level.

High-resolution structures of prions purified to homogeneity are beginning to emerge. In essence, they suggest that PrP^Sc^ assemblies are organized in a specific manner within a fibrillar supra-organization which is extremely stable. It remains so far difficult to conciliate or to find commonalities between the “deadpan” aspect of these emerging structures and PrP^Sc^ diversity and dynamicity. The next challenge will be to provide structures at high-resolution of the assemblies that preserve their diversity.
